# CRISPR-Cas12 Application for the Detection of *Pneumocystis jirovecii* in Immunodepression Patients Through Fluorescent and Lateral Flow Colorimetric Assay

**DOI:** 10.3390/ijms26178732

**Published:** 2025-09-08

**Authors:** Daniel Ulloa, Constanza Núñez, Romina Matamala, Aníbal San Martín, Dayana Páez-De Ávila, Jheyson Mercado-Vides, Juan Narváez, Juan Aguirre, Brian Effer, Isabel Iturrieta-González

**Affiliations:** 1 Doctoral Program in Sciences, Mention in Applied Cellular and Molecular Biology, Universidad de La Frontera, Francisco Salazar Ave. 01145, Temuco 4811230, Chile; d.ulloa09@ufromail.cl; 2 Biochemistry Program, Universidad de La Frontera, Francisco Salazar Ave. 01145, Temuco 4811230, Chile; c.nunez05@ufromail.cl (C.N.); r.matamala04@ufromail.cl (R.M.); 3 Biotechnology Program, Universidad de La Frontera, Francisco Salazar Ave. 01145, Temuco 4811230, Chile; a.sanmartin03@ufromail.cl; 4 Center for Genetics and Molecular Biology, Universidad del Magdalena, 29H3 Street No. 22-01, Santa Marta 470004, Colombia; dayanapaezpa@unimagdalena.edu.co (D.P.-D.Á.); sci.mvjj@gmail.com (J.M.-V.); jnarvaez@unimagdalena.edu.co (J.N.); jaguirrep@unimagdalena.edu.co (J.A.); 5 Center of Excellence in Translational Medicine, Universidad de La Frontera, Francisco Salazar Ave. 01145, Temuco 4811230, Chile; 6 Laboratory of Infectology and Clinical Immunology, Department of Preclinic Sciences, Medicine Faculty, Center of Excellence in Translational Medicine-Scientific and Technological Nucleus (CEMT-BIOREN), Universidad de La Frontera, Ave. Francisco Salazar 01145, Temuco 4811230, Chile

**Keywords:** CRISPR-Cas12, *Pneumocystis jirovecii*, lateral flow assay, diagnostic

## Abstract

Pneumonia caused by *Pneumocystis jirovecii* poses a serious threat, particularly to immunocompromised patients such as those with HIV/AIDS, transplant recipients, or individuals undergoing chemotherapy. Its diagnosis is challenging because current methods, such as microscopy and certain molecular tests, have limitations in sensitivity and specificity, and require specialized equipment, which delays treatment initiation. In this context, CRISPR-Cas12-based methods offer a promising alternative: they are rapid, highly specific, sensitive, and low-cost, enabling more timely and accessible detection, even in resource-limited settings. We developed a simple and rapid detection platform based on the CRISPR-Cas12 coupled with lateral flow strips. A guide RNA was designed against *DHPS*, *β-tubulin*, and *mtLSU rRNA* genes. The guide corresponding to *β-tubulin* showed high sensitivity in the detection of *P. jirovecii* to produce a detectable fluorescence signal within the first 20–30 min. In addition, it demonstrated high specificity for *P. jirovecii* when DNA from other microorganisms was used. When coupled with lateral flow strips, high sensitivity and specificity were also observed for detecting positive samples, without the need for genetic amplification. CRISPR-Cas12 successfully detected *P. jirovecii* infection in an initial diagnostic application, demonstrating the potential of this method for integration into public health diagnostic systems, particularly in field, due to its adaptability, speed, and ease of use.

## 1. Introduction

*Pneumocystis jirovecii* (ID:42068) is an opportunistic, non-cultivable, and ubiquitous fungus belonging to the phylum Ascomycota [1,2], which infects humans and can be potentially lethal in immunocompromised patients [3], primarily causing severe and potentially fatal pneumonia (PCP) (Huang et al., 2011), as well as other pulmonary diseases such as chronic obstructive pulmonary disease (COPD) [4].

Global estimates have reported 500,000 cases of PCP annually since 2013 [5]. Although the incidence has decreased with the implementation of highly active antiretroviral therapy (HAART), it remains elevated among patients without access to treatment, mainly in developing countries [6], ranging from 2% to 60% in AIDS populations [7]. In non-HIV immunocompromised patients, the high PCP rate is largely attributed to the increased use of immunosuppressants, higher doses, or combinations of different immunosuppressive agents, which increase susceptibility to pneumocystis infection [8,9], maintaining a high mortality rate [10].

Due to the difficulty of culturing *P. jirovecii* [4], diagnoses have traditionally relied on clinical symptoms and radiographic findings [11]. However, these are often nonspecific and can resemble other infectious agents; moreover, *P. jirovecii* may colonize the respiratory tract without manifesting any clinical or radiological features [12]. Consequently, laboratory detection remains essential, primarily via microscopy, which allows a direct visualization of the cystic or trophic forms of *P. jirovecii* in clinical samples—most commonly bronchoalveolar lavage (BAL) fluid [13], induced sputum [14], and, in more complex cases, lung biopsy [15]. Staining methods include calcofluor white, Giemsa, toluidine blue O (TBO), and Grocott–Gomori methenamine silver (GMS) staining [11].

Although microscopy remains the gold standard for *P. jirovecii* detection, it has limitations in sensitivity and specificity [16], as it depends on the skills and experience of the observer [17], as well as slower detection times and associated costs [18]. Calcofluor-white allows rapid visualization of cysts; however, it requires a fluorescence microscope [19,20]. Giemsa staining enables visualization of both asci and trophic forms depending on the pathogen load; however, low loads reduce sensitivity and specificity [21]. GMS staining can detect *P. jirovecii* in both samples of lung tissue and respiratory secretions, with high sensitivity and specificity [16], but it is more labor-intensive and costly than other stains [22]. TBO staining highlights the cystic forms of *P. jirovecii* but has lower sensitivity than other staining methods [20,21].

As a result, molecular assays for *P. jirovecii* detection have become increasingly common, including polymerase chain reaction (PCR) [23], loop-mediated isothermal amplification (LAMP) [24], and antibody-antigen assays [25], offering non-invasive testing with higher sensitivity [26]. Real-time PCR has markedly improved diagnostic sensitivity compared to conventional staining methods [16], achieving sensitivity levels 10^4^–10^6^ times higher [11]. This is because it enables quantification of low *P. jirovecii* loads, whereas microscopic examinations in non-HIV patients are prone to false negatives due to low fungal burden [12]. However, PCR cannot directly distinguish between past and active infections, as it detects pathogen DNA that may persist a long time after the infection resolution [27], and requires specialized laboratory facilities, thus limiting its applicability in primary care-particularly in resource-limited settings [28]. LAMP operates at relatively low temperatures, enabling accessible and user-friendly diagnosis, but its high contamination risk often leads to false positives in negative controls [29].

Therefore, there is a need to develop new, more accurate, quicker, and more cost-effective diagnostic tools for *P. jirovecii*. In recent years, metagenomic next-generation sequencing (mNGS) has been employed due to its speed and high detection rates [30]. However, its high cost and inability to differentiate colonization from infection can result in unnecessary overtreatment [12]. Another emerging approach is the implementation of CRISPR-Cas (clustered regularly interspaced short palindromic repeats associated with Cas proteins) technology, which has gained popularity in bio-detection due to its biocompatibility, unique cleavage mechanism, ease of operation, and straightforward design [31].

CRISPRs constitute an adaptive immune mechanism in bacteria and archaea, consisting of short repetitive sequences of 30–40 nucleotides alternating with spacers (remnants of bacteriophage sequences). These short sequences originate from viral genomes that previously infected these microorganisms [32]. They are believed to provide a form of genetic memory, as upon reinfection, these sequences are transcribed into CRISPR RNAs (crRNAs), which form functional complexes with Cas proteins (endonucleases) to lead them to the genetic material of the invading virus for degradation [33]. For the crRNA-Cas complex to cleave or degrade the invader’s genetic material, the crRNA must recognize a protospacer adjacent motif (PAM) sequence. Upon PAM recognition, the complex performs a precise cut, degrading the invader’s genetic material and thereby controlling the infection [34,35].

Cas12a (formerly Cpf1) belongs to Class 2 CRISPR systems, which consist of effector complexes with a single large, multidomain Cas protein capable of performing all required functions independently [31]. Cas12a recognizes thymine-rich PAM sequences and generates blunt-ended double-stranded breaks, known as “cis” cleavage [34]. It also provides a secondary domain capable of nonspecific single-stranded DNA (ssDNA) cleavage via “trans” activity [36]. This property has made it highly valuable for nucleic acid detection systems [35].

Cas12a-based detection systems primarily employ two approaches: fluorescence and lateral flow assays (LFA). In fluorescence-based detection, a ssDNA reporter molecule labeled with a fluorophore-quencher (FQ) pair generates a detectable fluorescent signal when Cas12a cleaves the reporter, releasing the fluorophore [37]. LFAs consist of a paper strip containing two bands: a test line with anti-rabbit antibodies and a control line with immobilized streptavidin [38]. When loading a sample in which the FAM-biotin probe has been cleaved by Cas12, biotin-labeled ssDNA is captured by streptavidin at the control line, while FAM-labeled ssDNA-nanoparticle complexes are captured by the anti-rabbit antibody at the test line, producing a color change in both lines to indicate a positive result [39].

In this context, the implementation of emerging technologies such as CRISPR-Cas12 has opened new possibilities in molecular diagnostics, offering significant advantages in specificity, speed, and adaptability compared to traditional methods. However, CRISPR-based diagnostics applied to fungal pathogens still face important limitations. These include a reduced sensitivity in the absence of a prior nucleic acid amplification step; the requirement for higher DNA concentrations to obtain reliable results in lateral flow formats compared to fluorescence assays; and the risk of false negatives when relying on a single guide RNA per target gene. These constraints underline the need for further optimization and adaptation of such platforms before their clinical implementation. Its ability to detect nucleic acids with high precision through fluorescent or colorimetric lateral flow readouts has proven particularly promising in clinical settings where early detection is critical for prognosis in immunocompromised patients. To enhance this diagnostic strategy, three highly conserved genes with recognized phylogenetic and diagnostic value have been selected: the mitochondrial large subunit ribosomal RNA (*mtLSU rRNA*) gene, the dihydropteroate synthase (*DHPS*) gene, and the β-tubulin gene. The *mtLSU rRNA* gene was included due to its presence in multiple copies within the mitochondrial genome, increasing detection sensitivity. The *DHPS* gene, although single-copy, encodes an essential enzyme in the folate biosynthesis pathway and is clinically relevant as both a diagnostic marker and a determinant of sulfonamide resistance. The *β-tubulin* gene encodes a structural protein of the cytoskeleton, highly conserved among fungi, but containing species-specific regions that allow the discrimination of *P. jirovecii*. These molecular targets enable specific pathogen identification, even at low loads, thus facilitating sensitive detection applicable to various clinical contexts. Therefore, the development of a CRISPR-Cas12-based lateral flow platform for *P. jirovecii* detection represents not only an innovative alternative, but also a potentially more accessible and efficient tool compared to standard techniques such as microscopy and real-time PCR-particularly in rural areas with limited access to highly sophisticated equipment or trained personnel.

Therefore, the development of a CRISPR-Cas12-based platform for *P. jirovecii* detection represents an innovative alternative to standard techniques such as microscopy and real-time PCR. In this study, we aimed to establish a laboratory-based diagnostic approach using two complementary readouts: fluorescence and lateral flow detection assays. We demonstrate that the fluorescence readout enabled highly sensitive detection with no more than 40 ng of DNA, while lateral flow testing provided robust positive results at 100 ng.

## 2. Results

### 2.1. Primers, gRNAs, and Reporter Probes

**Figure 1:**
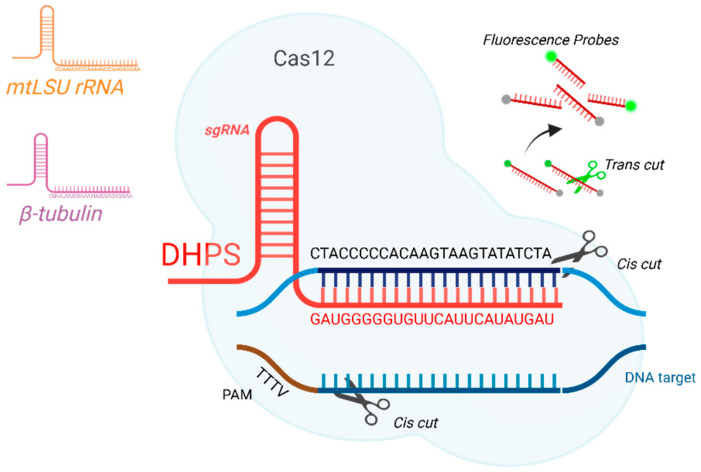
Schematic representation of target sequence detection using the CRISPR-Cas12 system. gRNA targeting sequence of the *DHPS* (red), *β-tubulin* (purple), and *mtLSU rRNA* gene (brown). After this, the “Cis” cut is activated (black scissors) in the DNA (blue). The cis cut activates the “Trans” activated cut (green scissors), responsible for breaking the probes that release fluorescence.

Table 1 summarizes the sequences of the primers, guide RNAs (gRNAs), and reporter probes used in this study. These were specifically designed and synthesized for the detection reactions employing the CRISPR-Cas12 system. In addition, Figure 1 illustrates the target sequence of each analyte to be detected.

### 2.2. Detection of P. jirovecii Through Microscopic Examination and PCR Amplification of the Respiratory Sample

**Figure 2:**
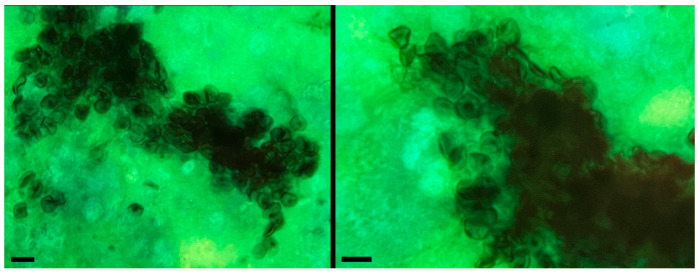
Cystic forms of *P. jirovecii* in respiratory samples stained with Gomori’s methenamine silver (magnification 1000×). Scale bar = 10 µm.

Microscopic examination of respiratory samples stained with Gomori’s methenamine silver and visualized by bright field microscopy revealed pleomorphic, rounded, or elliptical cystic forms of *P. jirovecii* in six out of seven samples analyzed, either as isolated structures or in clusters, as shown in Figure 2. Samples were considered negative when these structures were absent. All samples were also studied by PCR of the *mtLSU rRNA* region (Figure S1) as previously described [1]. As a positive control for the DNA extraction process, human β-globin amplification was also performed. The results were also correlated with clinical information of the patients, as all of them presented with respiratory symptoms such as fever or subfebrile temperatures, progressive dyspnea, and dry cough, suggestive of *P. jirovecii* infection, and chest CT images showing ground-glass opacities. One of the samples, corresponding to a pharyngeal wash, did not show cystic forms of the fungus in the microscopic examination; however, it was still considered positive because detection was achieved through the amplification of the *mtLSU rRNA* region.

### 2.3. Detection by Fluorescence Using CRISPR-Cas12

#### 2.3.1. Optimal DNA Amount by Fluorescence

Experimental assays were conducted to establish the optimal conditions for *P. jirovecii* detection using a CRISPR-Cas system coupled to fluorescence readout. Among the parameters evaluated were the target genes *β-tubulin*, *DHPS*, and *mtLSU rRNA*, using DNA amounts ranging from 0 to 100 ng to determine the optimal quantity of genetic material required to generate fluorescence through Cas12 activity.

**Figure 3:**
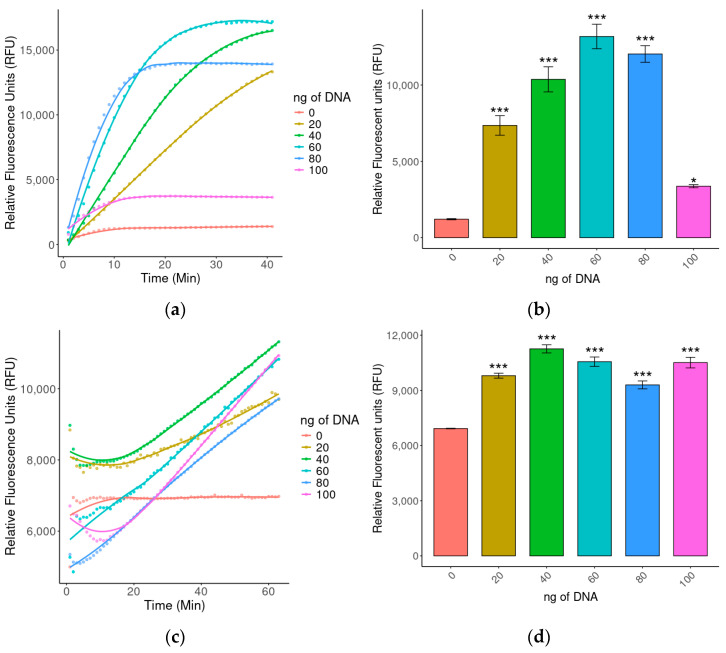

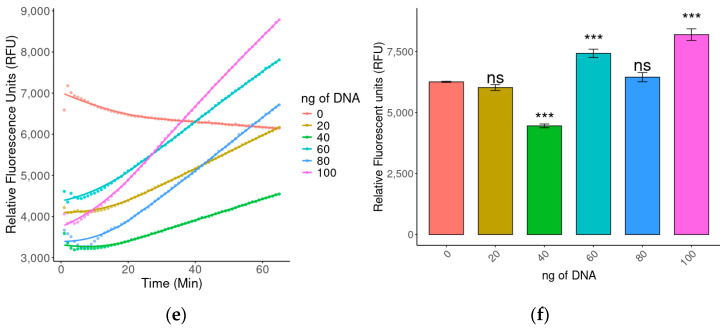
Evaluation of three target genes for fluorescence-based detection of *P. jirovecii*. (**a**,**c**,**e**) Fluorescence curves over time for *β-tubulin*, *DHPS*, and *mtLSU rRNA*, respectively, at different DNA amounts (0–100 ng); (**b**,**d**,**f**) Comparative boxplots of the maximum fluorescence achieved for *β-tubulin*, *DHPS*, and *mtLSU rRNA*, respectively, according to DNA amount. Statistically significant differences are indicated by asterisks (*: *p* < 0.05; ***: *p* < 0.001). No significant statatistical difference was denoted with ns.

As shown in Figure 3, the *β tubulin* gene exhibited the highest sensitivity for *P. jirovecii* detection, as evidenced by the stronger fluorescence signal obtained at lower DNA amounts, with 20 ng being sufficient to achieve detection within the first 20–30 min. In contrast, the *DHPS* gene required at least 40 ng to produce a detectable signal and remained less consistent overall. The *mtLSU rRNA* gene, on the other hand, yielded weak and unclear signals even at higher amounts and was therefore excluded as a diagnostic target.

#### 2.3.2. Specificity by Fluorescence

**Figure 4:**
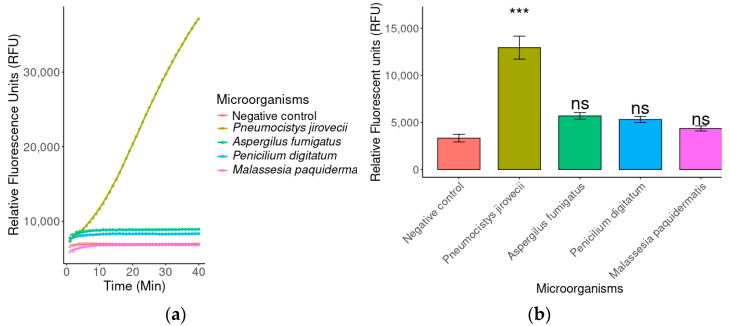
Comparison of fluorescence generated by *P. jirovecii* versus three other opportunistic pathogens and a negative control (water). (**a**) Fluorescence curve over time, showing a substantial and specific increase in the signal generated by *P. jirovecii;* (**b**) Bar graph showing the mean fluorescence reached at 20 min. Only the *P. jirovecii* sample exhibited significantly higher fluorescence (*** *p* < 0.001) compared with the negative control. No statistically significant differences (ns) were observed between the control and the other pathogens evaluated (*Aspergillus fumigatus*, *Penicillium digitatum*, and *Malassezia pachydermatis*).

The specificity of the detection system was assessed using DNA from other opportunistic pathogens, which, like *P. jirovecii*, are clinically associated with infections in immunocompromised patients-namely *A. fumigatus*, *P. digitatum*, and *M. pachydermatis*. As shown in Figure 4, just DNA from *P. jirovecii* produced a high, detectable fluorescence signal, whereas the other pathogens did not generate a significant signal.

#### 2.3.3. Fluorescence-Assisted Analysis for Biological Diagnosis

**Figure 5:**
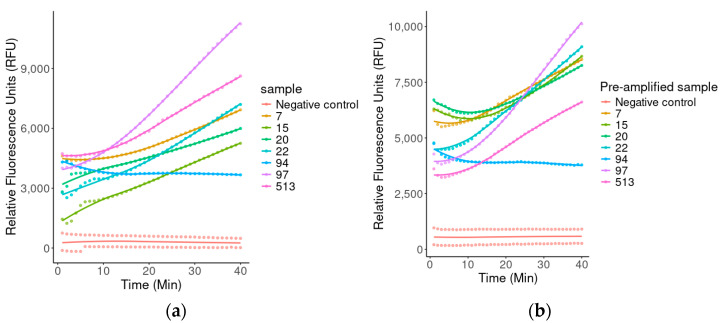
Comparison of fluorescence between the DNA extracted (**a**) and pre-amplified DNA (**b**) for seven positives patients by *P. jirovecii*, using the CRISPR-Cas12 system.

The sensitivity of the detection system was evaluated using both DNA and pre-amplified samples from seven *P. jirovecii*-positive patients. As shown in Figure 5, just samples 1, 2, 3, 5, 6, and 7 from *P. jirovecii*-positive patients generated a significantly higher fluorescence signal compared to their corresponding pre-amplified samples. Other samples displayed a more predictable behavior, also generating a significantly higher signal compared to the pre-amplified condition.

### 2.4. Detection by Lateral Flow Test

#### 2.4.1. Detection of the Optimal Amount of DNA

**Figure 6:**
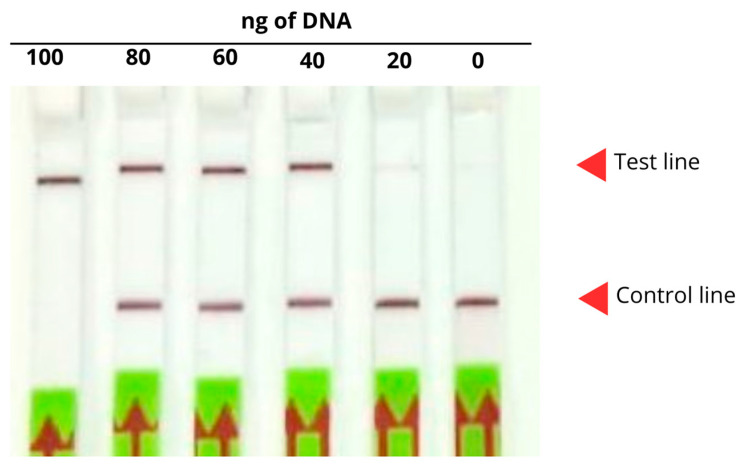
Lateral flow assay following the reaction with different amounts of *P. jirovecii* DNA (0–100 ng). The presence of a single band or two bands (red head arrow, test line, and control lines) indicates a positive diagnosis, whereas the presence single band (red arrow, control line) indicates a negative diagnosis.

Using different amounts of *P. jirovecii* DNA, the optimal quantity for detection using the CRISPR-Cas system coupled to the lateral flow assay was determined. Positive results were indicated by the presence of a band at the test line, or by bands at both the test and control lines, whereas negative results were identified by the presence of a band only at the control line. As shown in Figure 6, the optimal DNA amount for detection was 100–40 ng, as *P. jirovecii* could not be detected within the 20 ng range, where both bands were visible on the strip. Therefore, 100 ng was established as the minimum effective DNA amount for detection.

#### 2.4.2. Optimal Probe Concentration

**Figure 7:**
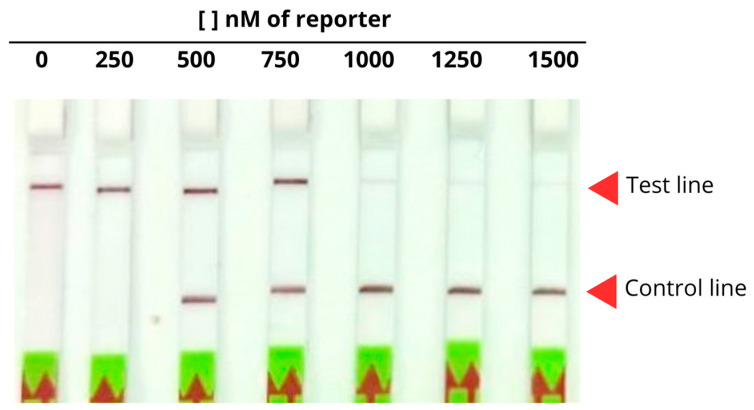
Visualization of lateral flow strips showing the effect of different concentrations of the FAM-Bio probe (0–1500 nM). The red-headed arrows indicate the test line and the control line, respectively.

Using a fixed DNA amount (100 ng) that belongs to *A. fumigatus*, different concentrations of the FAM-Bio probe were tested to determine the optimal amount for a clear negative result. As shown in Figure 7, higher concentrations (1000 and 1500 nM) resulted in the appearance of a control line band on the lateral flow strip, indicating a negative result. In contrast, lower concentrations (0 and 250 nM) yielded false positive results, with a clearly visible band at the test line. Therefore, a concentration of 1000 nM was determined to be the most efficient for the probe.

#### 2.4.3. Specificity in Lateral Flow Strips

**Figure 8:**
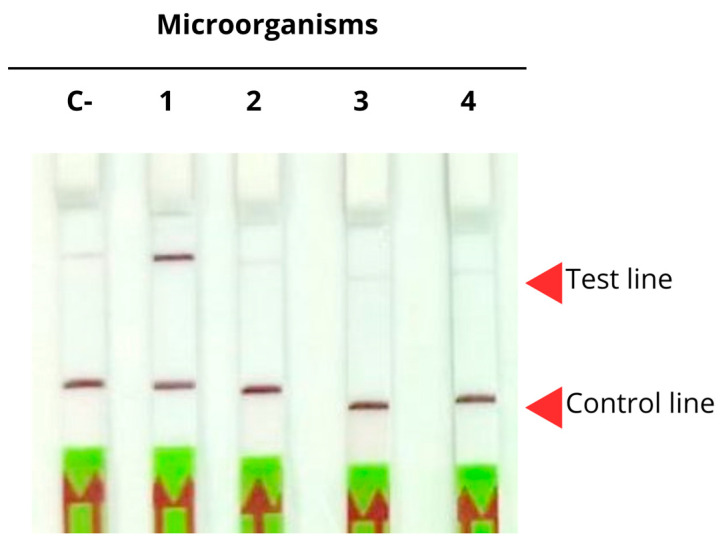
Lateral flow strips demonstrating the specificity of the CRISPR-Cas12 system in detecting DNA from different opportunistic pathogens: *P. jirovecii* (1), *A. fumigatus* (2), *P. digitatum* (3), and *M. pachydermatis* (4), negative control (C-).

To evaluate the specificity of the assay for *P. jirovecii* detection, the previous results were compared against other opportunistic pathogens, namely *Aspergillus fumigatus*, *Penicillium digitatum*, and *Malassezia pachydermatis*, as previously used in Section 2.3.2. As shown in Figure 8, just *P. jirovecii* yielded a positive result, whereas the samples corresponding to *Aspergillus fumigatus*, *Penicillium digitatum*, and *Malassezia pachydermatis* displayed bands on both lines, corresponding to negative results, thus confirming the high specificity of the assay.

#### 2.4.4. Optimal DNA Amount

**Figure 9:**
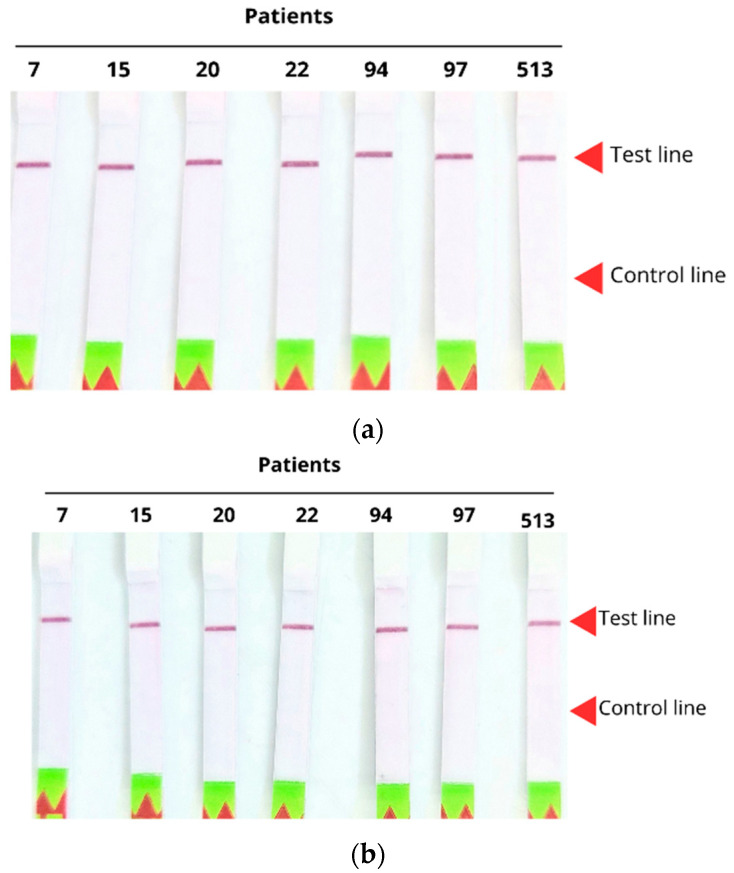
(**a**) Lateral flow strips detecting biological samples from seven patients using CRISPR-Cas12. Samples amplified by conventional PCR using primers specific to *β-tubulin*; (**b**) Non-amplified samples, directly obtained from the prior DNA extraction of the biological specimens.

Clinical samples from seven *P. jirovecii*-positive patients were evaluated under two conditions: with and without prior amplification of the genetic material. The results, shown in Figure 9, indicate that both conditions (Figure 9a,b) enabled successful detection, demonstrating that the system is sufficiently sensitive to detect the pathogen without the need for prior amplification.

## 3. Discussion

*P. jirovecii*, as a common opportunistic pathogen, represents a potentially life-threatening risk for immunocompromised patients. Accurate and early diagnosis is crucial for guiding treatment decisions and thereby contributing to patient recovery [11]. Conventional diagnostic methods, based on staining and microscopic examination of samples, display low sensitivity [22], thus highlighting the need for new diagnostic approaches based on molecular techniques such as conventional PCR (considered the gold standard) and ELISA assays, which offer higher sensitivity [40]. However, there is an urgent need for quick, cost-effective, and easy-to-interpret diagnostics that can be applied in situ, which provide results within minutes, and do not require sophisticated equipment. In this context, CRISPR-Cas12 technology emerges as a viable model for developing quick and versatile diagnostic kits [41].

In this research, we proposed the implementation of a novel and quick method for diagnosing *P. jirovecii* pneumonia in patients, based on the CRISPR-Cas12 system. This methodology, which relies on the capacity of the CRISPR-Cas12 complex to recognize and cleave DNA and the monitoring of this cleavage through fluorescence or lateral flow assays, also enables the identification of single-nucleotide polymorphisms (SNPs) [42], a critical feature for detecting clinically relevant strains or mutations, such as those conferring antibiotic resistance [43]. This technology has already been applied for the detection of specific pathogenic strains such as *Mycobacterium*, *Staphylococcus*, and SARS-CoV-2 [44], demonstrating its adaptability to detect multiple microorganisms, genotyping human DNA, and identifying mutations in circulating tumor DNA [45]. CRISPR has also been increasingly applied in molecular diagnostics, including for the African swine fever virus [46], human tuberculosis [47], and hepatitis B virus (HBV) using LAMP-Cas12a methodology, which provided quick and accurate results at low cost and without the need for specialized equipment [48]. Fungal pathogens such as *Candida albicans* [49] and *Aspergillus fumigatus* [50] have also been successfully detected, highlighting the versatility of this approach. Furthermore, CRISPR-Cas13 has been used for RNA detection [28], including one of the first studies aimed at detecting *P. jirovecii* RNA.

Unlike RNA detection with CRISPR-Cas13, DNA has a double-stranded structure, it is less prone to degradation [26], and does not require DNA-mediated RNA synthesis, thereby reducing total assay time and avoiding technical errors that could impact cost and result variability [45]. For these reasons, in this study, we designed and evaluated guide RNAs (gRNAs) targeting three DNA genes: *mtLSU rRNA*, *DHPS*, and *β-tubulin*.

The *mtLSU rRNA* gene (mitochondrial large subunit ribosomal RNA) has been widely used in molecular studies due to its high conservation [51] and its presence in multiple copies within the pathogen’s mitochondria, which enhances sensitivity in amplification-based methods [42]. Additionally, the sequence is exclusive to *P. jirovecii*, making it a reliable marker for differentiation from other fungi [52]. For these reasons, it is the most used target in qPCR and conventional PCR assays for clinical samples [53]. However, its high copy number also increases susceptibility to mutations in critical probe-binding sites, which can result in false negatives, as reported in some studies [51]. Despite being an ideal early-stage diagnostic target, in this study, the gRNA designed against *mtLSU rRNA* did not yield an adequate signal, consistent with observations by Zhan et al. [28] when detecting *mtLSU rRNA* RNA with Cas13, and with reports of false negatives in real-time PCR assays caused by point mutations such as C210T in the probe-binding region [51]. These results suggest that despite its high mitochondrial abundance and sensitivity, mutations may compromise detection, as shown previously, where fluorescence changes were not statistically significant compared to the negative control. Consequently, this target was deemed inviable for further evaluation in positive sample recognition.

In contrast, detection was more sensitive and efficient for *DHPS* and *β-tubulin* targets. This differs from some reports [28,54] where *mtLSU rRNA* was the main target, but multiple gRNAs were designed per target, yielding variable sensitivities. The low or null sensitivity observed here may be due to possible secondary structure formation in the gRNA, such as hairpin structures or increased complementarity to the target that interfere with CRISPR binding and cleavage efficiency, as noted by Liu Qiming et al. [26].

The *DHPS* gene encodes dihydropteroate synthase, a key enzyme in the folate biosynthesis pathway of *P. jirovecii* [55], and has been studied both as a diagnostic marker and for its role in sulfonamide resistance [56,57], the main treatment for this infection. Unlike *mtLSU rRNA*, *DHPS* is a single-copy gene, which reduces sensitivity when working with low DNA amounts [58]. However, its sequence specificity allows for differentiation of *P. jirovecii* from other microorganisms and offers the potential to detect resistant variants, adding clinical value in patients with poor treatment response [43]. In our results, the gRNA designed for *DHPS* produced a stronger detection signal than *mtLSU rRNA*, but required higher DNA amounts (~60 ng) to achieve positive detection, consistent with the lower sensitivity expected for single-copy genes compared with multicopy mitochondrial genes [58].

The *β-tubulin* gene encodes an essential structural protein in the microtubules of the cellular cytoskeleton. It is highly conserved among fungal species [59] but contains enough specific regions to differentiate between genera and species, including *P. jirovecii* [53]. Its stability, specificity, and demonstrated performance in amplification and quick detection assays [60] make it a reliable molecular marker, even under conditions of low DNA content or in contaminated clinical samples [61]. In our study, *β-tubulin* detection by fluorescence occurred within the first 15 min with high statistical significance (*p*  <  0.001), making it our primary study target. After optimizing detection parameters for this target, we evaluated the specificity of its gRNAs, which detected *P. jirovecii* exclusively and showed no cross-reactivity with other organisms, as shown previously.

An important methodological consideration for CRISPR-Cas12-based diagnostics is whether to include a prior DNA amplification step or to perform direct detection from raw samples [33]. Amplification, typically by PCR or isothermal methods such as RPA or LAMP, significantly enhances sensitivity, thus enabling the detection of very low amounts of genetic material. This is particularly valuable in cases of low pathogen loads, such as early infections or in immunocompetent patients. However, omitting amplification shortens assay time, reduces costs, and simplifies the workflow, which is especially advantageous in field diagnostics or resource-limited settings. Nonetheless, without amplification, sensitivity depends heavily on Cas12 reaction efficiency and initial DNA quantity [62]. In this study, we opted for pre-amplification to ensure robust results, but future optimizations may focus on direct detection without amplification, especially if more efficient Cas enzymes or DNA amount methods are implemented.

Unlike other studies using isothermal amplification methods such as RPA and LAMP [63,64], we used conventional Taq polymerase-based PCR kits, as this study represents an early stage aimed at validating diagnostic prototype performance and gradual clinical integration. Later stages will explore and report on isothermal amplification methods, which simplify processing and reduce reliance on specialized equipment, as successfully applied in *P. jirovecii* and other pathogen detection [28,45,47,65].

We evaluated samples from seven patients under two experimental conditions— with and without prior DNA amplification—using β-tubulin as the target. As previously shown, CRISPR-Cas12 detection without amplification was possible only in samples with high fungal loads, producing clear fluorescence signals within short times. Samples with low *P. jirovecii* loads displayed detectable but significantly weaker signals without amplification, suggesting that direct detection efficiency is strongly linked to initial DNA quantity. Incorporating a prior PCR amplification step enabled detection of all samples, regardless of fungal load, which confirms that this strategy significantly enhances assay sensitivity.

Lateral flow assay (LFA) results obtained within 30 min demonstrated lower sensitivity compared with fluorescence assays. This is because LFA relies on the accumulation of nanoparticle–antibody conjugates at the test or control lines [62], which requires enough gold nanoparticles to be visible [54]. In contrast, fluorescence changes are readily detected by instrumentation, which explains why higher DNA amounts were needed for conclusive LFA results, as shown in Figure 6, where the minimum required DNA amount for significant detection was doubled. Nevertheless, LFA maintains a robust diagnostic potential due to its ease of interpretation. Importantly, we confirmed assay specificity by discriminating *P. jirovecii* from other respiratory pathogens such as *A. fumigatus*, *P. digitatum*, and *M. pachydermatis*, supporting its application in clinical settings where cross-reactive microorganisms are common.

This study developed a basic proof-of-concept model, limited by the small sample size and the use of only one gRNA per target gene. However, the methodology demonstrates the feasibility of developing a quick and straightforward pneumonia diagnostic test, with the future challenge of achieving fully in situ detection directly from sputum, thus reducing turnaround times and increasing robustness. Future work could explore more efficient Cas proteins with higher nucleic acid detection sensitivity for field-deployable kits, alongside further reaction parameter optimization to produce qualitative results more rapidly.

The strategy of combining pre-amplification with Cas12a-mediated trans-cleavage improved both sensitivity and efficiency. Alternative methods such as LAMP or RPA could further reduce assay time, supporting timely clinical decision-making. However, quantitative metrics such as the minimum detectable copy number remain challenging, as the correlating pathogen load with infection versus colonization depends on methodology, infection stage, and patient population [52,66,67,68,69,70]. For example, *P. jirovecii* load in HIV-negative patients is generally lower than in HIV-positive patients, reducing positivity rates and complicating infection–colonization differentiation by qPCR [70]. In Piñana et al., qPCR results were confounded by overlapping Ct value thresholds for infection and colonization [70].

To improve the sensitivity of the assay, several strategies could be considered. First, the design and evaluation of multiple sgRNAs per target gene could increase recognition efficiency and reduce the risk of false negatives. Second, the integration of more efficient isothermal amplification methods, such as optimized RPA or LAMP protocols, would allow reducing the amount of input DNA required and shortening reaction times. Finally, further optimization of reaction conditions would contribute to improving the signal-to-noise ratio, enabling the detection of lower pathogen loads that are clinically relevant, particularly in HIV-negative patients.

## 4. Materials and Methods

### 4.1. Biological Samples

The *P. jirovecii* DNA was obtained from seven samples collected from adult patients diagnosed with HIV infection at the Hospital Dr. Hernán Henríquez Aravena. The samples were obtained through sputum (3), bronchoalveolar lavage (3), and pharyngeal wash (1), following prior approval by the Ethics Committee of the Servicio de Salud Araucanía Sur (protocol code No. 260, approved on 9 November 2021).

### 4.2. Primers, gRNAs, and Probes

Forward and reverse primers were designed against the target genes *DHPS* (MH753369.1), *β-tubulin* (EU979570.1), and *mtLSU rRNA* (KU744687.1), flanking the regions used for the design of the guide RNAs (gRNAs). gRNAs were designed using the online tool http://crispor.tefor.net, version 5.2 (Santa Cruz, CA, USA) (accessed on 23 July 2024), and specificity was evaluated using NCBI’s Primer-BLAST. Fluorescent reporter probes 5′6-FAM-TTTT TTTT TTTT-BHQ1 3′ (FAM-BHQ1) [47] and chromatographic probes 5′6-FAM-TTTT TTTT TTTT-Biotin 3′ (FAM-Bio) were synthesized by Integrated DNA Technologies, Inc. (Coralville, IA, USA). The EnGen Lba Cas12a (Cpf1) enzyme was obtained from New England Biolabs (Ipswich, MA, USA). For lateral flow assays, the HybriDetect kit (Milenia Biotec—GieBen, Germany) was used following the manufacturer’s instructions. All sequences were synthesized by IDT (S1). The gRNAs were aliquoted and stored at −80 °C until use.

### 4.3. Detection of P. jirovecii Through Microscopic Examination of the Respiratory Sample

Detection of *P. jirovecii* from respiratory samples was carried out through microscopic observation of the fungal cystic forms. Approximately 100 µL of the sputum sample was deposited and spread onto a microscope slide, covering an area of approximately 15 mm in diameter. For BAL and pharyngeal wash samples, prior centrifugation was performed at 5000 rpm for 5 minutes (min) to concentrate the cells, and the resulting pellet was used to prepare the smear. The smears were air-dried at room temperature and subsequently fixed with methanol (Merck, Darmstadt, Germany) for 2 min. Staining was performed using Gomori’s Methenamine Silver (GMS) method with the Methenamine silver plating kit acc. to Gomori (Sigma-Aldrich, St. Louis, MO, USA), following the manufacturer’s instructions. Finally, microscopic examination of the cystic forms was conducted using an Olympus CX21 microscope (Olympus, Volketswil, Switzerland) under 1000× magnification with oil.

### 4.4. Evaluation of Detection Using Fluorescence Coupled to CRISPR-Cas12

#### 4.4.1. Identification of the Optimal DNA Amounts

Detection assays were performed to determine the optimal DNA amounts detectable by the Cas12 enzyme. The target genes *DHPS*, *β-tubulin*, and *mtLSU rRNA* were selected for evaluation, using gRNAs specifically designed for each gene. In the initial step, the CRISPR-Cas12 reaction mix was prepared, consisting of 1 μL of gRNA (1 μM μL^−1^), 2 μL of 10× R2.1 buffer, 1 μL of Cas12 enzyme (1 μM μL^−1^), and 12 μL of ultrapure water to complete a final volume of 16 μL per reaction. The mixture was incubated at 25 °C for 10 min. Following incubation, 2 μL of FAM-BHQ1 probe (100 nM) was added, according to the protocol described by Ai et al. [47]. Subsequently, various amounts of DNA amplicons corresponding to the aforementioned genes (0, 20, 40, 60, 80, and 100 ng) were tested, adding 2 μL of each to reach a final reaction volume of 20 μL. A no-DNA control (sterile distilled water) was included as an internal negative control. Reactions were incubated at 37 °C for 40 min in a real-time thermocycler (Applied Biosystems StepOne 48, Foster City, CA, USA), with fluorescence monitored every minute. For subsequent evaluations, the gene showing the best performance in terms of sensitivity and fluorescence signal was selected.

#### 4.4.2. Evaluation of Specificity by Fluorescence

The specificity of *P. jirovecii* detection was assessed by comparison with other infectious agents that cause diseases in immunocompromised individuals, namely *Aspergillus fumigatus*, *Penicillium digitatum*, and *Malassezia pachydermatis*. For this purpose, the reaction protocol described in Section 2.4.1 was repeated, using DNA from the aforementioned pathogens while maintaining identical reaction and detection conditions, with fluorescence monitored at one-minute intervals.

#### 4.4.3. Diagnosis of Biological Samples by Fluorescence

Clinical samples from patients with confirmed *P. jirovecii* diagnosis were analyzed.

For this purpose, the DNA was extracted from each respiratory sample using the commercial Mini kit QIAamp DNA (Quiagen, Basel, Switzerland), following the manufacturer’s instructions. The DNA obtained was stored at −20 °C until the diagnosis by fluorescence. For each patient, two conditions were evaluated: one sample with the selected gene amplified by PCR using the primers described in Figure 1 and another non-amplified sample, both adjusted to 20 ng of DNA. The FAM-BHQ1 probe was used at a concentration of 100 nM. Detection was then performed using the real-time thermocycler method previously described in Section 2.4.1.

### 4.5. Detection by Lateral Flow Test Coupled to CRISPR-Cas12

#### 4.5.1. Determination of the Optimal DNA Amount

CRISPR-Cas12 reactions were prepared as described in the previous sections. Different amounts of *P. jirovecii* DNA (0, 20, 40, 60, 80, and 100 ng) were evaluated in a CRISPR reaction, as outlined in Section 2.4.1. After 30 min of incubation, 10 µL of the reaction mixture was combined with 80 µL of the buffer provided in the HybriDetect kit (Milenia Biotec, Germany). The end of the lateral flow strip was then immersed in this mixture, and the signal was visually interpreted within the first 5 min. A sample was considered positive when a colored band appeared at the test line (either alone or in combination with the control line), whereas the presence of a single band at the control line was interpreted as a negative result.

#### 4.5.2. Determination of the Optimal Probe Concentration

Using a fixed amount of target DNA at the highest fluorescence signal previously observed, different concentrations of the FAM-Bio probe (1500, 1250, 1000, 750, 500, 250, and 0 nM) were evaluated. Incubation and detection conditions were identical to those described in the previous section

#### 4.5.3. Evaluation of Specificity in Lateral Flow Strips

For the specificity assessment using lateral flow strips, DNA from *A. fumigatus*, *P. digitatum*, and *M. pachydermatis* (100 ng) was used, following the conditions described in Section 2.4.1.

#### 4.5.4. Evaluation of Sensitivity in Amplified and Non-Amplified Clinical Samples

Clinical samples from seven patients with confirmed *P. jirovecii* diagnosis were analyzed. For each patient, two conditions were evaluated: one PCR-amplified sample and one non-amplified sample, both adjusted to a final amount of 100 ng of DNA. Volumes varied according to extraction yield, and a FAM-Bio probe at a concentration of 1000 nM was used. Detection was subsequently performed following the lateral flow assay protocol previously described in Section 2.4.1.

#### 4.5.5. Diagnosis of Biological Samples Through Lateral Flow Strips

Clinical samples from patients with confirmed *P. jirovecii* diagnosis were analyzed. For each patient, two conditions were evaluated: one PCR-amplified sample and one non-amplified sample, both adjusted to 20 ng of DNA. A FAM-Bio probe at a concentration of 1000 nM was used. Detection was then performed using the real-time thermocycler method previously described in Section 2.4.1.

### 4.6. Statistical Analysis

For statistical comparison between experimental conditions and the negative control, a one-way analysis of variance (one-factor ANOVA) was performed, followed by Dunnett’s multiple comparison test. This approach enables the identification of statistically significant differences between multiple treatments or DNA amounts and a single reference group. Data were analyzed in R (version 4.5.0) using the multicomp package for Dunnett’s test and tidyverse for data processing and visualization. The response variable was the fluorescence intensity (FAM), obtained after activation of the CRISPR-Cas12 system against different DNA amounts. For each experimental condition, the mean and standard error of fluorescence were calculated from seven independent biological samples (patients), each measured in four technical replicates. These values were represented in bar plots, including error bars and statistical significance symbols *(p*  <  0.05: *, *p*  <  0.01: **, *p*  <  0.001: ***).

## 5. Conclusions

In conclusion, in this study, CRISPR-Cas12 successfully detected *P. jirovecii* infection in an initial diagnostic application, demonstrating the potential of this method for integration into public health diagnostic systems, particularly in field settings, due to its adaptability, speed, and ease of use. However, broader verification in prospective studies is required. Limitations include the design of only one sgRNA per target gene; improved performance is likely achievable with multiple gRNAs per gene. To enhance reliability, future studies should apply this technology to a larger patient cohort.

## Figures and Tables

**Table 1:** Sequence of oligos, gRNAs and probes employed for the CRISPR-Cas12 assay.

Target Gene	Oligo	Sequences
*mtLSU rRNA*	pAZ102-E	5′-GATGGCTGTTTCCAAGCCCA-3′
	pAZ102-H	5′-GTGTACGTTGCAAAGTACTC-3′
	gRNA_lsu	UAAUUUCUACUAAGUGUAGAUAAUUAUUAGAAGGGAGUAUGAGAG
*DHPS*	dhps_3	5′-GCGCCTACACATATTATGGCCATTTTAAATC-3′
	dhps_4	5′-GGAACTTTCAACTTGGCAACCAC-3′
	gRNA_dhps	UAAUUUCUACUAAGUGUAGAUGAUGGGGGUGUUCAUUCAUAUGAU
*β-tubulin*	tub_f	5′-TCATTAGGTGGTGGAACGGG-3′
	tub_r	5′-ATCACCATATCCTGGATCCG-3′
	gRNA_tub	UAAUUUCUACUAAGUGUAGAUCUAAUUUCGAAAAUUCGAGAGGAA
Probes	FAM_BHQ1	56-FAM/TT TTT TTT TTT T/3BHQ_1
	FAM_BIOTIN	56-FAM/TT TTT TTT TTT T/3Bio

## Data Availability

The sequences and new information created and used in this research are available to everyone.

## Supplementary Materials

The following supporting information can be downloaded at: https://www.mdpi.com/article/10.3390/ijms26178732/s1.
