# Supplementary material for: CRISPR-Cas12 Application for the Detection of Pneumocystis jirovecii in Immunodepression Patients Through Fluorescent and Lateral Flow Colorimetric Assay
Source: Int J Mol Sci. 2025 Sep 8;26(17):8732. doi: 10.3390/ijms26178732 (PMC12429702; doi:10.3390/ijms26178732)
Supplement: Supplementary file 1 [file ijms-26-08732-s001.zip › ijms-3846725-supplementary.pdf]

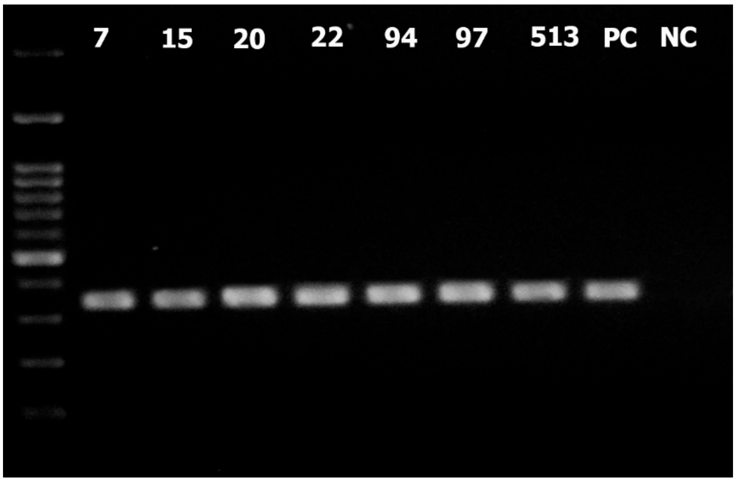

Figure S1. Amplification of patients sample against mtLSU rRNA gen from *P. jirovecii*. PC: Positive control (genomic DNA belong to *P. jirovecii*). NC: Negative control (All reagent were included except DNA).
